# Dynamic prediction model of fetal growth restriction based on support vector machine and logistic regression algorithm

**DOI:** 10.3389/fsurg.2022.951908

**Published:** 2022-09-23

**Authors:** Cuiting Lian, Yan Wang, Xinyu Bao, Lin Yang, Guoli Liu, Dongmei Hao, Song Zhang, Yimin Yang, Xuwen Li, Yu Meng, Xinyu Zhang, Ziwei Li

**Affiliations:** ^1^ Faculty of Environment and Life Sciences, Beijing University of Technology, Intelligent Physiological Measurement and Clinical Translation, Beijing International Base for Scientific and Technological Cooperation, Beijing, China; ^2^ Department of Obstetrics, Peking University People’s Hospital, Beijing, China

**Keywords:** fetal growth restriction, FGR, dynamic prediction, prediction model, multiple gestational weeks

## Abstract

**Background:**

This study analyzed the influencing factors of fetal growth restriction (FGR), and selected epidemiological and fetal parameters as risk factors for FGR.

**Objective:**

To establish a dynamic prediction model of FGR.

**Methods:**

This study used two methods, support vector machine (SVM) and multivariate logistic regression, to establish the prediction model of FGR at different gestational weeks.

**Results:**

At 20–24 weeks and 25–29 weeks of gestation, the effect of the multivariate Logistic method on model prediction was better. At 30–34 weeks of gestation, the prediction effect of FGR model using the SVM method is better. The ROC curve area was above 85%.

**Conclusions:**

The dynamic prediction model of FGR based on SVM and logistic regression is helpful to improve the sensitivity of FGR in pregnant women during prenatal screening. The establishment of prediction models at different gestational ages can effectively predict whether the fetus has FGR, and significantly improve the clinical treatment effect.

## Introduction

Fetal growth restriction (FGR) is one common complication of pregnancy and accounts for increasing perinatal morbidity partly (1). FGR refers to fetal growth that has not reached its genetic potential due to maternal, fetal, and placental pathological factors. Typically, FGR is characterized by fetal ultrasound estimates of weight or abdominal circumference lower than the 10th percentile for the same gestational age (2). The onset of FGR is subtle, and it can only be diagnosed at the time of delivery, which brings great difficulties to prevention and treatment, and is accompanied by a variety of complications that are difficult to identify. In 2013, the Royal College of Obstetricians and Gynaecologists in the United Kingdom and the American College of Obstetricians and Gynaecologists summarized multiple methods for predicting FGR (3). Domestic and foreign scholars generally focus on exploring the change of FGR-related detection parameters (4–10) and the prospective prediction of FGR (5, 12–18). However, these are based on the physiological or pathological static parameters at a certain moment to predict whether pregnant women will suffer from FGR in the next stage, which cannot fully reflect the real dynamic physiological status of the human system.

Pregnancy is a dynamic process, and various physiological and pathological parameters are changing at different gestational stages. Therefore, to accurately assess the risk of FGR, it is necessary to combine a variety of FGR risk factors and comprehensively evaluate the status of pregnant women at different gestational weeks to establish a dynamic prediction model for FGR.

## Materials and methods

### Subjects and specimens

124 pregnant women were enrolled. 64 cases with FGR were case group (FGR group), and the other 60 cases without any pregnancy complications were control group (normal group). Fetal FGR was defined as a fetal birth weight less than the 10th percentile of the same gestational age. Retrospective methods were used to collect the maternal medical records of pregnant women at different gestational weeks. The basic information about pregnant women is shown in Table 1.

**Table 1:** Basic information about the pregnant women.

Parameters (Unit)	FGR group	Normal group
Number	64	60
Age (years)	31.4 ± 3.8	30.3 ± 2.6
Delivery gestational age (weeks)	38.8 ± 1.35	39.6 ± 1.0
Height (m)	1.61 ± 0.05	1.63 ± 0.05*
Pregnancy weight (kg)	53.8 ± 6.16	55.0 ± 7.68
Pregnancy BMI (kg/m^2^)	20.7 ± 2.15	20.5 ± 2.57

Note: *P < 0.01.

At present, most of the studies on the risk of FGR are based on the detection and assessment of single factors, and there is a lack of comprehensive studies on multiple risk factors and predictors of FGR, which cannot accurately diagnose fetal FGR during pregnancy. The epidemiological factors, blood pressure factors, and biochemical factors including gestational weight, uterine height, abdominal circumference, systolic blood pressure, diastolic blood pressure, and hemoglobin were obtained at 14–19 weeks, 20–24 weeks, 25–29 weeks and 30–34 weeks of gestation. Fetal factors affecting FGR at 20–24 weeks and 25–29 weeks of gestation were also obtained, including estimated term ideal weight, ultrasound parameters, estimated fetal weight, and the difference between the 10th percentile of fetal ideal weight. An independent sample t-test was used to test the influence of these risk factors on FGR. As shown in Table 2, this study selected easily accessible clinical predictors, which were all high-risk factors for FGR with statistical differences.

**Table 2:** Summary of risk factors related to fetal growth restriction.

Type	Risk factors of FGR
Epidemiological factors	Height, gestational weight gain, age,uterine height, abdominal circumference, adverse pregnancy history, pregnancy complicated with uterine fibroids
Fetal factors	TOW,ultrasound parameters (BPD, HC, AC, FL), EFW, EFW-fetal weight 10th

Notes: gestational weight gain, The difference between the end-of-pregnancy weight and the pre-pregnancy weight; abdominal circumference, The length of a circle around the abdomen along the horizontal line of the umbilicus is measured with a soft tape measure; TOW, estimate term optimum weight; BPD, biparietal diameter; HC, head circumference; AC, abdominal circumference; FL, femur length; EFW, ultrasound estimated fetal weight; EFW-fetal weight 10th,estimated difference between fetal weight and fetal curve 10th.

According to previous studies, TOW was 40 weeks (280 days) of Term Optimal Weight (in g) (19), and the formula for TOW is shown in Formula 1.


(1) TOW=3405.6+17.726×(gest−280)+8.245×(height−162.7)+6.406×(weight−65.1)+114.3(ifparity≥1)−65.7(if24≤BMI<26.9)

In the formula, “gest” refers to the days of delivery for pregnant women(term delivery, 259 to 294 days of gestation, 37 to 42 weeks of gestation), and “height” and “weight” refer to the value of the pregnant woman at the first visit of the ultrasonic examination during the middle gestational age. When parity ≥1, the yield adjustment coefficient was 114.3; otherwise, it was 0. When 24 ≤ BMI < 26.9, BMI is −65.7; otherwise, BMI is 0.

In this study, the fetal weight estimation Formula (2), recently published by INTERGROWTH-21st, was used to estimate the actual intrauterine weight of the fetus at different gestational weeks (20).

(2) $$Ln(EFW)=\;\;5.084820-54.06633\times (\frac{AC}{100}{)}^{3}+3.136370\times (\frac{HC}{100})-95.80076\;\times log\;(\frac{AC}{100})\times (\frac{AC}{100}{)}^{3}$$

where HC is uterine height, AC is abdominal circumference and EFW is estimated fetal weight.

Fetal weight ratio 10th percentile curve equation (19):

$$\;\mathrm{fetal}\;\mathrm{weight}\;10\mathrm{th}=435.8-44.89\times \mathrm{GA}+1.502\times GA^{2}-0.01495\times GA^{3}\;$$

GA is gestational age.

In this study, two methods were used to construct FGR prediction models at 14–19 weeks, 20–24 weeks, 25–29 weeks, and 30–34 weeks of gestation. The first method: We chose the Libsvm toolbox as the pattern recognition classifier. At different gestational weeks, FGR risk factors with statistical differences were selected as the input of SVM classifier (21), and an effective risk assessment model of FGR was obtained after training. The second method: At different gestational weeks, all the parameters affecting FGR were analyzed by multi-factor Logistic regression (22), and the regression equation was obtained as shown in Formula (4), based on which the prediction model of FGR was established as shown in Formula (5).


$$\mathrm{lo}g_{it}\left(p\right)=\ln\;\frac{\;p}{1-p}\;$$

$$P=\frac{1}{1+e^{-\left(\alpha +\beta_{1}x_{1}+\beta_{2}x_{2}+...+\beta_{i}x_{i}\right)\;}}$$

### Model analyses

In this study, FGR risk prediction models were established by support vector machine (SVM) and multivariate logistic regression at 14–19 weeks, 20–2weeks, 25–29 weeks, and 30–34 weeks of gestation. The SVM algorithm is especially suitable for the small sample size of the included study but with many variables, and is very accurate in the ability to simulate complex nonlinear decision boundaries (23). When the outcome is classified data, logistic regression analysis can be used to study the quantitative impact of the joint action of multiple factors and their interaction to the outcome.

Firstly, when the SVM was used to establish the FGR prediction model, the characteristic parameters with statistical differences affecting FGR at 14–19 weeks, 20–24 weeks, 25–29 weeks, and 30–34 weeks of gestation were selected as the input parameters of the model training. In each gestational period, 75% of the sample data were randomly selected as the training set and the remaining 25% as the test set. There were 93 cases of data in the training set and 31 cases of data in the test set. For 93 cases of data in the training set, we used the SVM method and cross-validated it to train the prediction model. The performance of the optimal model was finally selected by testing the data of the test set, and the accuracy of the test set was obtained.

**Figure 1:**
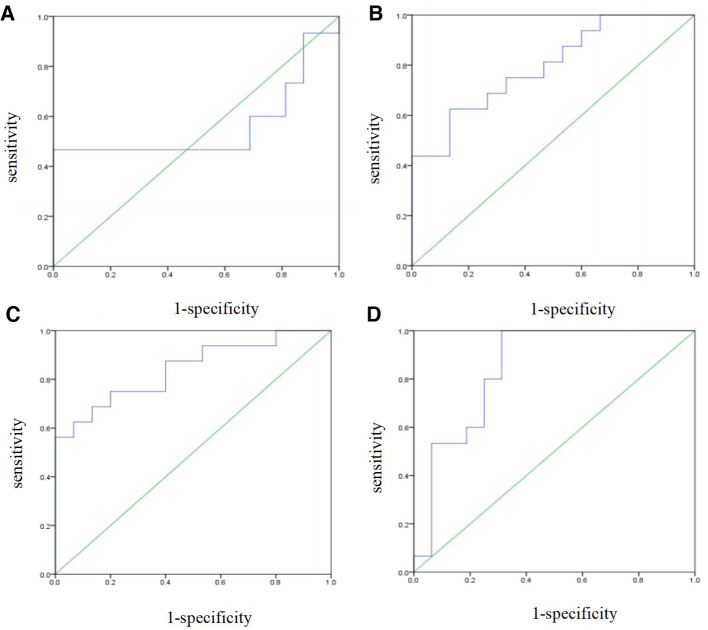
FGR prediction model was established by support vector machine. (**A**) ROC curve of the risk assessment model of FGR at 14-19 gestational weeks. (**B**) ROC curve of the risk assessment model of FGR at 20∼24 gestational weeks. (**C**) ROC curve of the risk assessment model of FGR at 25∼29 gestational weeks. (**D**) ROC curve of the risk assessment model of FGR at 30∼34 gestational weeks.

The prediction performance of the support vector machine prediction model for each gestational week was evaluated, and the results were shown in Table 3 below. The ROC curve of the influence of related factors on FGR disease was shown in Figure 1.

**Table 3:** Performance evaluation of SVM-based disease prediction models for FGR at various gestational weeks.

Models	AUC [95%CI]	*P*	AC	SE	SP
Week14∼19	0.558 [0.331,0.786]	0.580	0.548	0.467	0.625
Week20∼24	0.796 [0.641,0.950]	0.005	0.710	0.750	0.667
Week25∼29	0.842 [0.702,0.981]	0.001	0.742	0.750	0.733
Week30∼34	0.846 [0.701,0.990]	0.001	0.774	0.800	0.750

Note: AUC, the area under ROC curve; AC, accuracy; SE, sensitivity; SP, specificity. *P* < 0.05 was statistically significant.

Secondly, when using multivariate logistic regression to establish the FGR prediction model, the dependent variable was the fetus being diagnosed as being smaller than its gestational age. Multivariate logistic regression was performed for the parameters of epidemiological factors measured at 25–29 weeks and 30–34 weeks of gestation. Based on the analysis results, the regression equation was obtained, and then the FGR prediction model was established based on the related factors at 14–19 weeks, 20–24 weeks, 25–29 weeks, and 30–34 weeks of gestation. Among them, the FGR prediction model of 14–19 weeks of gestation is:

$$P=\frac{1}{1+e^{-m}},m=\;-2.037X_{1}-3.28X_{2}-3.097X_{3}+4.609X_{4}+0.174X_{5}$$

If the pregnant woman is 35 years old, X_1 _= 1; otherwise, X_1_ = 0; if the pregnant woman has adverse pregnancy history, X_2 _= 1, otherwise, X_2 _= 0; X_3_ = 1 if the pregnant woman suffers from pregnancy complicated with uterine fibroids, otherwise X_3_ = 0; X_4_ = height of the pregnant woman (*m*); X_5 _= weight gain of pregnant women (kg).

The prediction model of FGR at 20–24 weeks of gestation was as follows:


$$P=\frac{1}{1+e^{-m}},\;m=-3.304X_{1}-26.913X_{2}-81.720X_{3}+3.520X_{4}17-0.295X_{5}-0.011X_{6}-1.114X_{7}\;17+3.422X_{8}+8.844X_{9}+5.626X_{10}-0.172X_{11}17-0.030X_{12}$$

If the pregnant woman is 35 years old, x_1 _= 1; otherwise, X_1 _= 0; if the pregnant woman has adverse pregnancy history, X_2 _= 1, otherwise, X_2_ = 0; X_3 _= 1 if pregnant woman suffers from pregnancy complicated with uterine fibroids, otherwise X_3_ = 0; X_4 _= height of pregnant woman (*m*); X_5_ = weight gain of pregnant women (kg); X_6 _= TOW (g); X_7 _= BPD (cm); X_8 _= HC (cm); X_9 _= AC (cm); X_10 _= FL (cm); X_11 _= EFW (g); X_12_ = (EFW−fetal weight 10th) (g).

The prediction model of FGR between 25 and 29 weeks of gestation was as follows:

$$P=\frac{1}{1+e^{-m}},\;m=-3.411X_{1}-5.477X_{2}-9.443X_{3}+4.369X_{4}-0.064X_{5}-0.009X_{6}-2.614X_{7}\;+0.914X_{8}+4.002X_{9}-0.933X_{10}-0.035X_{11}-0.002X_{12}-0.483X_{13}-0.005X_{14}\;$$

where, if the pregnant woman is 35 years old, X_1 _= 1, otherwise X_1 _= 0; if the pregnant woman has adverse pregnancy history, X_2 _= 1,otherwise the X_2 _= 0; X_3_ = 1 if pregnant woman suffers from pregnancy complicated with uterine fibroids, otherwise X_3_ = 0; X_4_ = height of pregnant woman (*m*); X_5 _= weight gain of pregnant women (kg); X_6 _= TOW (g); X_7 _= BPD (cm); X_8 _= HC (cm); X_9 _= AC (cm); X_10 _= FL (cm); X_11 _= EFW (g); X_12 _= (EFW−fetal weight 10th) (g); X_13_ = Uterine height (cm); X_14_ = abdominal circumference (cm).

The prediction model of FGR at 30–34 weeks of gestation was as follows:

(9) $$P=\frac{1}{1+e^{-m}},\;\;\;m=-2.085X_{1}-3.804X_{2}-4.325X_{3}+23.937X_{4}17-0.171X_{5}-0.007X_{6}-0.319X_{7}+0.027X_{8}$$

where, if the pregnant woman is 35 years old, X_1 _= 1; otherwise, X_1_ = 0; if the pregnant woman has adverse pregnancy history, X_2 _= 1, otherwise, X_2_ = 0; X_3_ = 1 if pregnant woman suffers from pregnancy complicated with uterine fibroids, otherwise X_3_ = 0; X_4 _= height of pregnant woman (*m*); X_5_ = weight gain of pregnant women (kg); X_6 _= TOW (g); X_7 _= Uterine height (cm); X_8_ = abdominal circumference (cm).

**Figure 2:**
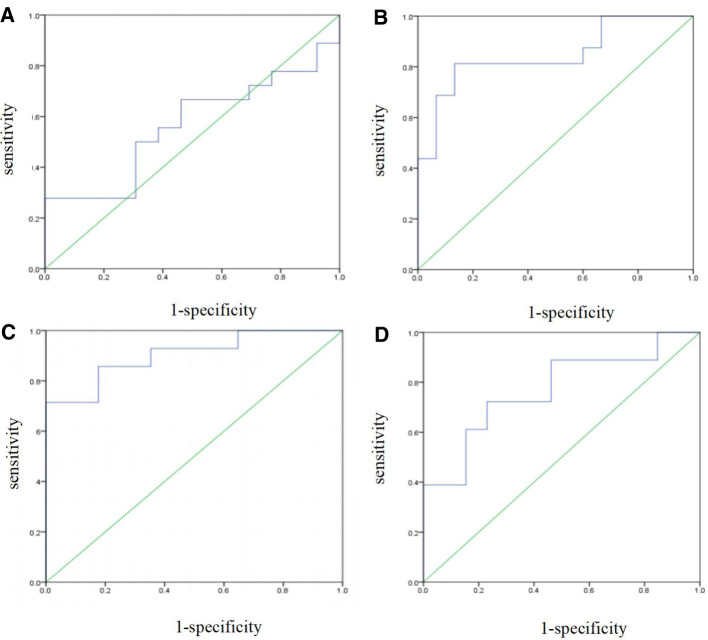
FGR prediction model was established by multivariate logistic regression. (**A**) ROC curve of the risk assessment model of FGR at 14–19 gestational weeks. (**B**) ROC curve of the risk assessment model of FGR at 20∼24 gestational weeks. (**C**) ROC curve of the risk assessment model of FGR at 25∼29 gestational weeks. (**D**) ROC curve of the risk assessment model of FGR at 30∼34 gestational weeks.

The prediction performance of the multi-factor Logistic regression prediction model at each gestational age was evaluated, and the results were shown in Table 4 below. The ROC curve of the influence of related factors on FGR disease was shown in Figure 2.

**Table 4:** Evaluation of FGR disease prediction models at various gestational weeks based on multivariate logistic regression.

Models	AUC [95%CI]	*P*	AC	SE	SP
Week 14∼19	0.846 [0.704,0.988]	0.548	0.484	0.333	0.692
Week 20∼24	0.796 [0.641,0.950]	0.001	0.839	0.813	0.867
Week 25∼29	0.903 [0.792,1.000]	0.000	0.839	0.857	0.824
Week 30∼34	0.846 [0.701,0.990]	0.012	0.710	0.611	0.846

Note: AUC, the area under the ROC curve; AC, accuracy; SE, sensitivity; SP, specificity. *P* < 0.05 was statistically significant.

We compared the results of the two methods for predicting FGR in four types of gestational age. According to the results in Tables 3, 4, the prediction accuracy of the two methods at 14–19 weeks of gestation was less than 60%. The SE of FGR predicted by SVM method was 0.467, and it predicted by multivariate logistic method was 0.333, indicating that the ability of predicting FGR correctly by these two models was not strong at this gestational age. At 20–24 and 25–29 gestational weeks, the accuracy (AC), sensitivity (SE) and specificity (SP) of the prediction model established by the multivariate logistic method were better than those of the SVM method. The results showed that the ability of adding fetal parameters to predict FGR from 20 to 30 weeks was stronger. And it is more suitable for multivariate logistic regression to predict. At 30–34 weeks of gestation, the sensitivity of SVM prediction model was 0.80, which was higher than that of multivariate logistic regression prediction model (0.611). Moreover, the specificity of the prediction model using SVM was 0.75, which was lower than 0.846 obtained by the multivariate logistic method, indicating that the ability of the multivariate logistic method to correctly determine the final pregnant women with FGR was low, but the ability to determine the non-FGR was high.

### Model validation

In order to verify the eight FGR prediction models obtained by using two methods in this study, 15 pregnant women (F1-F15) who were eventually diagnosed with FGR and 15 normal pregnant women (N1-N15) without pregnancy complications and complications were randomly selected as model test objects. The basic information is shown in Table 5 below. The SVM and multivariate logistic regression were used to verify the model function of pregnant women in four gestational stages. The verification results are shown in Table 6.

**Table 5:** Basic information of subjects in the validation model.

Basic Information (Unit)	N1-N15	F1-F15
The number of	15	15
Age (years)	30.1 ± 2.4	29.9 ± 2.5
Height (M)	1.64 ± 0.06	1.63 ± 0.05
Pre-pregnancy weight (kg)	54.3 ± 6.4	56.0 ± 7.0
BMI before pregnancy (kg/m^2^)	20.2 ± 2.1	21.0 ± 2.2

**Table 6:** Model validation results of fetal growth restriction.

Model function verification results	SVM				Multiple factors logistic			
	AUC [95%CI]	AC	SE	SP	AUC [95%CI]	AC	SE	SP
Week 14∼19	0.604 [0.399,0.809]	0.567	0.733	0.400	0.516 [0.302,0.729]	0.533	0.545	0.526
Week 20∼24	0.796 [0.641,0.950]	0.767	0.867	0.667	0.911 [0.805,1.000]	0.867	0.867	0.867
Week 25∼29	0.804 [0.643,0.966]	0.767	0.867	0.667	0.862 [0.732,0.993]	0.767	0.750	0.786
Week 30∼34	0.853 [0.716,0.990]	0.733	0.800	0.667	0.840 [0.698,0.982]	0.800	0.765	0.846

Note: AUC, the area under the ROC curve; AC, accuracy; SE, sensitivity; SP, specificity. *P* < 0.05 was statistically significant.

The data of 30 people were re-selected to perform functional verification of FGR classification prediction using SVM and multivariate logistic regression. The models of 20–24 weeks, 25–29 weeks and 30–34 weeks of gestation can have a good identification effect on FGR pregnant women, and the prediction effect of the model is the best in 20–24 weeks of gestation.

## Results

According to the comparison of the results, the ability of the two methods to predict FGR at 14–19 weeks of gestation was not high as the pregnancy progressed. The models of 20–24 weeks, 25–29 weeks, and 30–34 weeks of gestation had strong classification ability. The results of multivariate logistic regression were better in 14–19 weeks, 20–24 weeks, and 25–29 weeks, and the results of SVM regression were better in 30–34 weeks. The research results confirm the research significance of this topic.

## Discussion

FGR has been a hot topic of discussion and research among obstetricians because of its many complications. Ultrasonography, uterine height, and abdominal circumference are often used to comprehensively evaluate and predict FGR in clinical practice (7–9). Cordina M, Bhatti S et al. demonstrated that the average height, age, and weight of pregnant women with FGR were lower, and the paper also mentioned evidence that high fetal hemoglobin at the placental level was associated with the possibility of FGR (5). We aim to effectively predict whether a fetus is delivered with FGR or not. This requires us to analyze a variety of factors, pay attention to the changes in different physiological parameters, and establish FGR risk prediction models for different gestational weeks so that the assessment can be real-time and targeted.

The dynamic prediction model in this study can obtain the risk factors affecting FGR according to the gestational age of pregnant women, and select models of different gestational ages for prediction. It is simple, fast, and easy to promote, and has important clinical significance.

## Conclusion

In this study, statistically significant risk factors were included at 14–19 weeks, 20–24 weeks, 25–29 weeks, and 30–34 weeks of gestation by SVM and multivariate logistic regression, respectively, to classify whether neonates had FGR, and establish a dynamic model to predict FGR along with gestation. It is helpful to improve the sensitivity of prenatal screening for FGR in pregnant women and to prevent and treat it in advance. We hope that future studies on predicting FGR can be conducted in large, multicenter, multi-ethnic populations.

## Data Availability

The original contributions presented in the study are included in the article/supplementary materials, further inquiries can be directed to the corresponding author/s.
